# Comment on Jameson* et al.*: Prevalence of Antibodies against Hantaviruses in Serum and Saliva of Adults Living or Working on Farms in Yorkshire, United Kingdom

**DOI:** 10.3390/v6093415

**Published:** 2014-09-12

**Authors:** Jan Clement, Paula McKenna, Valentijn Vergote, Marc Van Ranst

**Affiliations:** 1 National Reference Centre for Hantavirus Infections, Laboratory of Clinical Virology, University Hospitals Gasthuisberg, Leuven, 3000, Belgium; E-Mails: valentijn.vergote@uzleuven.be (V.V.); marc.vanranst@uzleuven.be (M.V.); 2 Janssen, Pharmaceutical Companies of Johnson & Johnson, Beerse, 2340, Belgium; E-Mail: pmckenna@its.jnj.com

**Keywords:** Seoul virus (SEOV), hantavirus, wild rat, prevalence, risk factors, hemorrhagic fever with renal syndrome (HFRS), U.K., Northern Ireland

## Abstract

This British hantavirus IgG prevalence study, aimed at 119 asymptomatic farmers in England, and using indirect immunofluorescence assay (IFA) as screening technique, concluded that rat-transmitted *Seoul virus* (SEOV) might be the main suspect as hantaviral pathogen in the UK. Exactly the same conclusion, using the same IFA screening technique, resulted from a 1994 serosurvey in the same country, and in 627 clinical cases plus 100 healthy controls. SEOV-positive study subjects were also mainly farmers with heavy rat-exposure, but residing in Northern-Ireland, a region where all other known rodent reservoirs for pathogenic hantaviruses are known to be absent, except the wild rat. A rodent capture action in and around the farms of eight seropositives confirmed SEOV seropositivity in 21.6% of 51 rats. All SEOV seropositives were patients, hospitalized with an acute feverish condition, a majority of which having the clinical picture of hantavirus-induced nephropathy, known as hemorrhagic fever with renal syndrome (HFRS). Leptospirosis, often mimicking perfectly HFRS, was serologically excluded. Thus, SEOV was established as a human hantaviral pathogen in the UK and in Europe 20 years ago.

## 1. Introduction

We read with interest the study of Jameson *et al.* [1] concerning hantavirus IgG antibodies in serum and saliva of healthy farmers in Yorkshire, United Kingdom. This study raises a series of questions as to the scope and relevance of the study design, the methodology used, and the ensuing conclusions.

*Saaremaa virus* (SAAV) is once again cited as a hantavirus species circulating in Europe, and “confirmed to cause human disease within Europe” [1]. Hitherto, not a single biomolecularly confirmed SAAV case has been reported, and SAAV morbidity rates, let alone mortality rates, have often been mixed up with other subspecies of *Dobrava virus* (DOBV) cases, namely DOBV-Aa [2]. There is a growing agreement today amongst the global hantavirus expert community to consider SAAV only as one of the four subspecies of DOBV, and causing no or only subclinical human disease [3,4].

The authors Jameson* et al.* mention that rodents, together with insectivore species, intermittently excrete infectious (hanta) virus in urine, saliva and feces. To our knowledge, this has not yet been proven for insectivores, a fact of considerable importance for global epidemiology.

The authors cite two prior hantavirus IgG serosurveys in the U.K., one 1990 study in 320 asymptomatic Northern-Irish farmers, and one 1999 study in 606 asymptomatic English subjects, again farmers, farm workers and their families. The most important British hantavirus serosurvey so far, carried out by P. McKenna* et al.* [5,6] in 627 Northern-Irish hospitalized patients, and 100 healthy controls, is not however cited, despite the fact that the same screening technique, indirect immunofluorescence assay (IFA), was used on samples gathered between 1988 and 1992. In contrast to the current English study however, IFA was performed on a battery of nine different live hantavirus species, including two different *Seoul virus* (SEOV) strains, three different *Puumala virus* (PUUV), three then recently isolated DOBV strains, and the Korean prototype strain *Hantaan virus* (HTNV) 76-118. Moreover, IgM IFA and ELISA was also carried out on IgG positive samples, a missed opportunity for the current English study, since we know that a vast majority of hantavirus infections (up to 80% in Scandinavian PUUV infections [2]) can occur sub-clinically, or at least paucisymptomatically.

In the Introduction, it is stated that “it was not until 2012 that a causative hantavirus species was confirmed (in the U.K.)”. Since this statement refers to a hemorrhagic fever with renal syndrome (HFRS) case, serologically but not bio-molecularly proven to be a SEOV case [7], it should be put in due historical perspective. McKenna* et al.* showed already 20 years before that SEOV was by exclusion the only possible known causative agent in 16 Northern-Irish cases, of which 15 were hospitalized for fever and various acute reasons compatible with HFRS, e.g., 10/15 (66.6%) with unexplained acute renal failure (ARF), originally suspected for leptospirosis, mimicking HFRS [6]. Screening for leptospirosis however, appeared negative. Moreover, an acute recent hantavirus infection was proved in most IgG positive cases by IgG titers declining within weeks or months, and by positive IgM µ-capture ELISA in 8/14 sera tested subsequently. Lack of IgM positivity in six acute cases was probably due to the general lack of a SEOV antigen in the then available ELISA formats, offering at that time only two screening antigens, HTNV and PUUV. However, IFA IgG positivity was found in 15 out of 727 (2.1%) sera, with an almost exclusive reactivity against a Chinese SEOV rat strain R22. Sole reliance upon the then classic non-rat-derived screening antigens (*i.e.*, HTNV and PUUV), used at that moment for all other European hantavirus serosurveys, would have resulted in the detection of only 2/16 (12.5%) of positive cases in IFA IgG. Interestingly in comparison to the current study, one asymptomatic farmer from the control group was even IgM positive.

In contrast, however with the current study, no Northern-Irish IgG positive serum reacted against the three PUUV strains, used for the 1994 study. This was not surprising, since North-Ireland was and is the only region in West-Europe (together with Portugal and limited areas in the South of Norway and Sweden), where the PUUV reservoir *Myodes glareolus* (bank vole) is totally absent. Likewise, *Apodemus flavicollis* (yellow-necked mouse), the reservoir of the subspecies DOBV-af, is also absent in the whole Ireland. Moreover, since the HTNV and the SAAV reservoir *Apodemus agrarius* (striped field mouse) is equally absent in the entire U.K. (as in the rest of West-Europe), serologic cross-reactions with all these important pathogens (arvicoline PUUV, and murine DOBV or HTNV) could already be excluded 20 years ago. Thus, the pioneer 1994 study of McKenna* et al.* should still be considered as the first clinical series of 16 seroproven SEOV HFRS cases in the U.K., and even in Europe altogether, since this study fulfilled all the inclusion criteria now required by Center of Disease Control (CDC), Atlanta, GA, USA: suggestive clinical presentation, rising IFA IgG titers and positive IgM ELISA results.

Also in the Introduction, the authors coin the cited Yorkshire farmer with SEOV (Humber) infection “an autochthonous HFRS case”. In Scotland and England, at least two previous autochthonous HFRS cases after heavy rat exposure were described, one in Scotland in a boating-pond attendant with leptospirosis [8], and one in a boy playing in a Nottingham scrapyard infested with rats [9]. Both were diagnosed by positive IFA hantavirus serology, but not confirmed bio-molecularly, nor by PRNT, as in fact is also the case for the current Humber and Cherwell clinical cases [7,10].

## 2. Results

Many questions rise also as to the methodology and interpretation of this IgG serosurvey.

It is not clear why the authors, using a commercial IFA format, decided to quantify their results by a subjective gradation from − to +++, rather than using the highest dilution still giving characteristic intra-cytoplasmic fluorescence as classic end-point titer. This is in line with the recommendations of the commercial firm (Euroimmun) itself, allows a better spread of the obtained results from zero to 1/32,000, and has in fact been applied by the same authors for the serodiagnosis of their prior clinical cases, infected by Humber, respectively Cherwell SEOV [7,10]. Is, for instance, a +++ result equal to 1/32,000, or could it be 1/10,000 as well? This question is particularly relevant for the interpretation of the many possible cross-reactions, as observed: in fact, in seven out of nine seropositives in the current study.

To our knowledge, the current English study is the first British seroprevalence study showing, in a commercial screening format with six different hantaviral antigens, two clearly PUUV positive samples, out of nine seropositives,* i.e.*, 22%. This is in contradiction however with the results obtained 24 years earlier, but with the same screening technique and in the same research institute, then called Special Pathogens Reference Laboratory (SPRL), Porton Down, by Graham Lloyd, using HTNV and PUUV IFA for screening occupational groups at risk. In these groups, consisting of farm workers, water sporters, sewage workers, and Scottish nature conservancy workers, the overall positive serotype was PUUV, whereas a mixture of PUUV or SEOV/HTNV seropositives was only found in 18.9% of animal laboratory personnel. Remarkably, of the 130 examined farm workers, 21.5% was described to be only PUUV-positive [11,12]. Although of paramount importance in a European perspective, this second ever indication of PUUV presence in Britain is not discussed by the authors, except for an attempt to exclude a highly improbable American *Sin Nombre Virus* (SNV) infection. Performing rodent capture actions around the premises of positive study subjects, as was performed also 20 years ago [13], and in particular around the homes of the two PUUV positive subjects, would add perhaps most welcome insights in the infection mechanics of this by far most important hantaviral pathogen in Europe, of which virtually nothing is known so far in Britain [14]. Finally, a possible cross-reaction with another arvicoline pathogen,* Tula virus* (TULV), is not considered. When it is clear that the classic rodent reservoir for TULV,* Microtus arvalis* (common vole), is absent from Britain (except the Orkney Islands and Guernsey), the same is not true for the very common Eurasian water vole *(Arvicola amphibius*, formerly* A. terrestris*), which has been also shown to carry TULV [15], infecting forestry workers, even in a non-endemic area of East-Germany [16].

The risk factor analysis given on Table 2 [1] cannot be used as a general indication for incurring hantavirus infections in the U.K., given the implicit selection bias (only farmers) and a questionnaire focused too exclusively on one single hantavirus infection,* i.e.*, rat-transmitted SEOV. Moreover, seropositivity for both SEOV and PUUV was used indiscriminately in this risk assessment study (Table 2), thus further skewing results. If indeed the working hypothesis was “seeing rats”, then it is not clear why the odds ratio of this evident question was given as “non-assessed” (NA) on Table 2, seemingly in contrast to the information given in the Discussion, which suggests that 92.4% of the questionnaire respondents confirmed regularly seeing rats on their residence. Of note, this is in accordance with the study of *McKenna et al.*, in which the vast majority of SEOV positives were farmers with frequent rat exposure on their premises. Adding other, easily obtainable, but formerly proven relevant questions [17] to the questionnaire could have benefitted the further impact of this pioneering British HFRS risk analysis. For instance, after the first 1992–1993 Franco-Belgian PUUV outbreak, Van Loock* et al.* [18] found smoking a very significant risk factor (odds ratio (OR) 9.8, 95% confidence interval (CI) 2.6–36.6), confirmed 11 years later by a bigger Finnish study [19,20]. Hand-to-mouth transmission of infectious virus (and diminished barrier function of the lung as *porte d’entrée* of hantavirus?) might be enhanced by smoking, and is an almost daily risk in farming, much less so in many other professions. Moreover, and after the second 1996 Franco-Belgian PUUV outbreak, Crowcroft* et al.* [21] demonstrated a very high OR (19.4, CI 1.2–308.2) for the interaction of simply living <50 m from a forest and seeing rodents at home, *p* = 0.04. Forests, the preferred habitat of different hantavirus rodent reservoirs, and the risk of living near, or working in forests, have never been examined so far in a British risk analysis.

The authors depict a map of Yorkshire and the Humber, showing geographical distribution of the nine positive samples in six of the 34 surveyed districts, together with the localization of the two previous SEOV cases, and call it in their Discussion “a widespread rural circulation (of SEOV) in the region”. Without rodent studies in the same area, such an early assumption should be issued with extreme caution, since (A) it is derived from two clinical cases and nine seropositives in a limited IgG prevalence study on a single professional group at risk,* i.e.*, farmers with a presumably higher exposure to rats than to other rodent hantavirus carries; and (B) two of the nine seropositives (Table 2) were in fact infected with PUUV, not with SEOV (Table 1) [1].

Conversely, the same assumption was justified two decades earlier, whereby 16 SEOV seropositive clinical cases were detected in a very limited area in Northern-Ireland. Moreover, this human prevalence study was followed immediately (1994) by a capture action of a total of 141 rodents, solely around or in farms of eight SEOV positives, all living in County Down [13]. County Down is also a coastal region, at risk for floods, and with a port city (Bangor), potentially harboring imported rats. Out of 51 rats captured in County Down, 11 (21.6%) were found to have IFA IgG antibodies against the R22 strain,* i.e.*, the same strain to which the Northern-Irish cases had overwhelmingly reacted. Interestingly, 19/59 house mice (*Mus domesticus*) were also positive, much more so with HTNV 76-118 than with R22 sceening. Even more intriguing, seroprevalence was significantly higher in house mice captured inside, than outside the farms (*p* = 0.08), and lung antigen in these mice reacted strongly with convalescent sera from the locally infected farmers. These mice results were equivocal, and probably only cross-reactive, since the very common *Mus domesticus* is not a known reservoir of pathogenic hantaviruses. However, this puzzling finding in a prior British study of rodents on farms was worth a word of consideration in the current study, more so since the authors found a significant but unexplained reduction in risk of seropositivity (OR 0.006, *p* > 0.001), apparently by seeing mice indeed during the day on their farms (Table 2) [1]. If house mice on the British Isles (or perhaps only in North-Ireland?) were confirmed in subsequent studies to carry a HTNV-like pathogen, frequent sighting of such carriers cannot corroborate a lower human prevalence of a predominantly HTNV-like infecting agent, but it could explain perhaps the predominantly HTNV-like IFA results in the current study.

Indeed, it is not clear, nor is it explained in the Discussion, how the authors reached their final verdict as to the true nature of the infecting agent. When admittedly only plaque reduction neutralization tests (PRNT) could give a possible confirmation, it is striking to see that with the current Euroimmun SEOV screening antigen (the Korean urban rat strain 80-39), strong cross-reactions were observed with another murine screening antigen HTNV 76-118, to the point that the authors concluded in only one sample for a convincing SEOV infection, whereas in six other samples, a double serodiagnosis HTNV/SEOV was considered, because of an equal (1/9), or even stronger (regrettably not exactly titered) result with HTNV than with SEOV in 5/9 (55.5%) cases. The article nevertheless is almost exclusively dedicated to one single putative hantavirus pathogen (SEOV). In summary, 55.5% of the “positive” samples react more strongly with a heterologous than with an autologous murine screening antigen, and additionally 2/9 (22%) samples pertain in fact to an infection with a totally different arvicoline hantavirus species (PUUV). Hence, no valid conclusions can be drawn as to a new or better understanding of hantavirus epidemiology in the U.K. Given the overall negative IFA results with a third murine screening antigen, DOBV (in the Euroimmun IFA kit, apparently strain Slo_Af-BER), and more importantly, given the fact that the DOBV-Af reservoir *A. flavicollis* has only a patchy distribution in England and Wales south of the Wirral, a DOBV infection in the Yorkshire famers can admittedly be excluded. In summary, with these unusual murine-like results, a final diagnosis of a SEOV infection (and consequently a preponderant role of the wild rat as a source of infection in England) is not warranted without further confirmatory tests both in humans and in local rats. Moreover, the same marked HTNV/SEOV cross-reaction was noted in prior Humber, resp. Cherwell clinical cases, showing both ≥ 1:10,000 IFA IgG titers [7,10]. In contrast, the almost exclusive and high-titered SEOV results found in 1994 by McKenna* et al.* with the same screening technique, but with a different and sensitive Chinese wild rat screening antigen R22, leaves little doubt as to the infecting pathogen, which moreover confirmed the clinical data compatible with HFRS in cases, needing a hospitalization. In fact, only 1/15 samples in the McKenna* et al.* study showed an IFA IgG titer one dilution higher for HTNV than for SEOV.

## 3. Discussion

The idea that SEOV might be a pathogen “introduced recently to the region” (Discussion), perhaps via wharf rats from the ports on the Humber estuary [7], is maybe immediately intuitive, but must be confronted with the long history of hantavirus research: right after the isolation in 1982 of SEOV in Korea [22], this new pathogen was found virtually everywhere when it was looked after: in the New World, the Old World, and even in Africa, where up to very recently, hantaviruses were (incorrectly) claimed as being totally absent. The reason was and still is very simple: the SEOV rodent carrier, the brown rat, is the only global hantavirus reservoir, present everywhere. Thus, in the pioneer time of American hantavirology, several SEOV isolates were found in rats caught in port cities as Philadelphia (1984, Girard Point strain), Belem (1985 Brazil strain), New Orleans (1986 Tchoupitoulas strain), Baltimore strain (1987),* etc.* [23]. However, by far the earliest of all SEOV isolates (and even of hantavirus isolates altogether) in the West were performed by Graham Lloyd from 1977 through 1983, finding no less than five isolates from SEOV-infected rat immunocytomas, sent in from Belgium to PHE, Porton Down. He coined these isolates with the respective names given in French at the University of Louvain, Belgium, to the rat immunocytomas (IR),* i.e.*, IR33 up to IR490 [24]. These SEOV isolates survived storage at −70 °C for prolonged periods of up to ten years, and are probably still all available at PHE nowadays. Indeed, one of these, IR461, was sent in 1995 to the University of Belfast, where genetic characterization finally followed in 2003, *i.e.*, at least 24 years after its first isolation in February 1979 in PHE. IR461 was confirmed as being SEOV, but against all expectations, appeared not related to any known SEOV derived from laboratory rats [25]. The conclusion of Shi* et al.* was that IR461 resulted most likely from an introduction, in Belgium [26] or elsewhere in Eurasia, of a wild rat strain into a colony of laboratory rats.

If now, about 35 years after the first isolation of a SEOV in the U.K., recently two other SEOV strains, Humber [7] and Cherwell [10], were isolated again in PHE, and appeared surprisingly similar, but not identical, to IR461, there is no new phylogenetic argument, from a purely bio-molecular point of view, suggesting a recent introduction in the region of a “new” pathogen. The recent severe clinical presentation of SEOV nephropathy induced by Humber or Cherwell in England and Wales is moreover not a really new factor of concern: let us remember that the four PHE staff members, infected in 1977 with IR461, needed hospitalization for “varying degrees of respiratory problems, proteinuria, oliguria, and ARF during 2–3 weeks, two needing even acute hemodialysis” [24,27], whereas the 1997 Nottingham SEOV case needed even 17 days of peritoneal dialysis and two successive renal biopsies [9]. The rodent reservoir in these several lab rat, wild rat or pet rat infections is not essentially different, since remaining basically the same rodent species, *Rattus norvegicus.* Thus, if genetically, clinically and mammalogically there are no sizable differences between these three forms, there is no evidence-based reason to maintain further an artificial schism between lab rat, wild rat or pet rat SEOV species. After all, in the SEOV dendrogram produced after the Cherwell isolation [10], not only IR461, Humber and Cherwell found themselves together in the same (U.K.) clade, but the same was observed for SEOV strains of three totally different countries, being 80-39 (Chinese urban rat), Tchoupitoulas (USA wild rat) and Sapporo (Japanese laboratory rat) [10].

Finally, and perhaps most importantly, the so-called “resolving of the uncertainty surrounding the presence of a hantavirus in the U.K.”,* i.e.*, by finding SEOV (Discussion) has to be tempered, again with the often forgotten lessons of past hantavirus research. The current British hantavirus research situation is highly reminiscent of a similar situation in the USA in the early eighties, when seropositive wharf rats were sought and found in 1982 in seven international American port cities [28]. The several ensuing SEOV isolations (see here above) and repeated demonstrations in wild rats of SEOV infection rates of up to 57.7%, particularly in Baltimore, Maryland [29], was a source of excitement for the just born American hantavirus research, and led to high-running assumptions, for instance a never proven (but often cited) theory that chronic but asymptomatic *Baltimore rat virus* infection could lead to end-stage renal failure with hypertension, thus necessitating chronic hemodialysis [23,30]. However, the clinical impact of these early discoveries proved in the long run to be deceivingly low, leading to the recognition and seroconfirmation of only five SEOV HFRS cases in the USA, in an observation period of over 30 years so far [23]. This exceedingly low incidence is remarkably comparable to the current situation in the U.K., where after the initial 1994 description of 16 SEOV Northern-Irish cases, not a single British SEOV case was reported until 2013, except for the pediatric 1997 Nottingham case. Lack of medical awareness is the most probable explanation, since a straightforward explanation was initially not found for the combination of ARF with thrombocytopenia during the former laboratory outbreaks in Belgium, the UK and The Netherlands, nor even for the later 2011 Cherwell SEOV case in the English pet rat breeder [10]. In France, a hantavirus infection was initially not considered in the first bio-molecularly proven SEOV case in a pregnant woman with rapidly progressing renal involvement, and led to an urgent delivery by cesarean section [31,32]. Today, except for some rare warnings to the contrary [33,34], most hantavirus reviews still persist to state that SEOV HFRS is absent in the West, and moreover, that HFRS is even absent altogether in the New World, despite the local presence of millions of potentially SEOV-infected wild rats. Still nowadays largely ignored, the first (1993) clinical hantavirus cases in the new World, were HFRS cases, not so-called hantavirus pulmonary syndrome (HPS) cases. They were presenting with ARF and thrombocytopenia associated with leptospirosis, after heavy rat exposure due to local floods in Recife, Brazil. However, they were proven in IFA and ELISA to be in fact SEOV-induced, whereas leptospirosis was serologically excluded [35]. Subsequent reports in the Americas of predominantly HTNV-like serosurveys, or even of PRNT-proven domestic Baltimore SEOV clinical cases [34] were likewise ignored in later literature, after the exciting first (1994) description of HPS [36], and the characterization of a new autochthonous American hantaviral pathogen, *Sin Nombre virus* (SNV).

Let us hope that the current suddenly blooming British research on SEOV could at least stimulate worldwide a renewed interest in this virus, paradoxically the only global hantavirus pathogen, potentially present everywhere, known but neglected for more than three decades.
